# Conditional Deletion of the V-ATPase a2-Subunit Disrupts Intrathymic T Cell Development

**DOI:** 10.3389/fimmu.2019.01911

**Published:** 2019-08-13

**Authors:** Theodore V. Peterson, Mukesh K. Jaiswal, Kenneth D. Beaman, Joseph M. Reynolds

**Affiliations:** ^1^Center for Cancer Cell Biology, Immunology and Infection, Chicago Medical School, Rosalind Franklin University of Medicine and Science, North Chicago, IL, United States; ^2^Edward Hines, Jr. VA Hospital, Hines, IL, United States

**Keywords:** V-ATPase, thymocyte development, lymphopenia, a2V, Notch1, positive selection

## Abstract

Proper orchestration of T lymphocyte development is critical, as T cells underlie nearly all responses of the adaptive immune system. Developing thymocytes differentiate in response to environmental cues carried from cell surface receptors to the nucleus, shaping a distinct transcriptional program that defines their developmental outcome. Our recent work has identified a previously undescribed role for the vacuolar ATPase (V-ATPase) in facilitating the development of murine thymocytes progressing toward the CD4^+^ and CD8^+^ αβ T cell lineages. Vav1^Cre^ recombinase-mediated deletion of the *a2* isoform of the V-ATPase (a2V) in mouse hematopoietic cells leads to a specific and profound loss of peripheral CD4^+^ and CD8^+^ αβ T cells. Utilizing T cell-restricted Lck^Cre^ and CD4^Cre^ strains, we further traced this deficiency to the thymus and found that a2V plays a cell-intrinsic role throughout intrathymic development. Loss of a2V manifests as a partial obstruction in the double negative stage of T cell development, and later, a near complete failure of positive selection. These data deepen our understanding of the biological mechanisms that orchestrate T cell development and lend credence to the recent focus on V-ATPase as a potential chemotherapeutic target to combat proliferative potential in T cell lymphoblastic leukemias and autoimmune disease.

## Introduction

Thymic development of T lymphocytes occurs via irreversible progression through distinct developmental stages, ensuring a diverse, responsive, yet selective peripheral T cell compartment. Defects in this process contribute to a number of syndromes including immunodeficiencies, malignancies, and autoimmunity. Incredible efforts toward diagnosing the underlying mechanisms which govern progression through the checkpoints and distinct stages that define intrathymic T cell development have revealed key molecular players working in concert to shepherd progenitor cells along the T lineage pathway (1, 2). Yet it remains poorly understood how the intrinsic cell biology of developing thymocytes participates in the coordination of signals guiding developmental transition, and how these pathways might be targeted to affect the many deleterious outcomes of aberrant T cell development.

Thymocyte development begins as hematopoietic progenitor cells emigrate from the bone marrow into circulation, settle in the thymus, and adopt the T cell lineage developmental program. Phenotypically, thymocytes pass though several distinct developmental stages grossly delineated by CD4 and CD8 expression, and more precisely defined by the expression of CD44 and CD25, as well as other markers (3). Thymocytes first progress though four double negative (DN1-4; CD4^−^ CD8^−^) stages, develop into double positive (DP; CD4^+^ CD8^+^) cells, and subsequently undergo positive and negative selection. Cells that successfully complete this process emigrate from the thymus into peripheral circulation and lymphoid tissues as single positive CD4^+^ or CD8^+^ T cells. Notch1 signaling governs the earliest events in this progression and is absolutely required for progenitor cell commitment and further development of T cells (4, 5). However, while Notch1 is required during the DN3 stage (CD44^−^CD25^+^) at the first developmental checkpoint of β-selection, its influence then wanes as thymocytes shift to TCR-mediated gene networks for final development and selection (6–8). Identification of mechanisms by which cells orchestrate these shifting networks of developmental control remains an area of intense study.

V-ATPase is an essential proton pump which regulates pH in the cytoplasm and intracellular compartments of single- and multi-cellular organisms (9). V-ATPase often localizes to intracellular membranes and organelles, where acidification of the Golgi apparatus and vesicles in both the endocytic and secretory pathways depends on its function. Ligand-receptor dissociation, concentration of hormones within vesicles, and maintenance of enzymatically favorable pH within pathway compartments are some of the more generally accepted functions of V-ATPase in this regard, and perturbations in these housekeeping processes can have devastating effects (10–13). In mammals, the a subunit of the membrane-associated V_o_ domain exists in four isoforms and is preferentially expressed in distinct tissues and sub-cellular membranes (14). Selective targeting of the *Atp6v0a2* isoform (a2V) to early endosomes suggests a potential role in regulating critical membrane trafficking pathways (15, 16).

In this report, we show that conditional deletion of a2V in hematopoietic cells surprisingly leads to a profound deficiency of CD4^+^ and CD8^+^ αβ T cells in secondary lymphoid organs, though notably B cells, γδ T cells, and major myeloid lineages are present and appear developmentally unaffected. We traced this deficiency to events during intrathymic T cell development, and found that deletion of a2V affects this process during both the DN and DP stages. These T lineage-specific effects appear to be in part tied to abnormal control of Notch1 receptor processing and signaling, as well as perturbations in TCR-mediated selection and developmental processes. This phenotype demonstrates an unexplored function of the V-ATPase during T cell development, and opens new avenues of research into key events of lymphopoiesis.

## Materials and Methods

### Mice

a2V^fl/fl^ mice on the C57BL/6 background were generated as previously described (17). We crossed a2V^fl/fl^ mice with Cre-expressing strains from Jackson Laboratories (Vav1^Cre^ (stock number 008610), Lck^Cre^ (stock number 003802), and CD4^Cre^ (stock number 022071)) to conditionally delete a2V within the hematopoietic compartment or within developing thymocytes. Presence of the a2V^fl/fl^ gene was confirmed by PCR utilizing the primer pair 5′ AGGGTGGTGTCCTTTCACTCT 3′ and 5′ ATCCCCAGGATCCACGCAT 3′. Presence of the respective Cre transgene was confirmed utilizing the following primer pairs: *Vav1Cre*, 5′ AGATGCCAGGACATCAGGAACCTG 3′ and 5′ ATCAGCCACACCAGACACAGAGATC 3′; *LckCre*, 5′ TGTGAACTTGGTGCTTGAGG 3′ and 5′ CAGGTTCTTGCGAACCTCAT 3′; *CD4Cre*, 5′ GCGGTCTGGCAGTAAAAACTATC 3′ and 5′ GTGAAACAGCATTGCTGTCACTT 3′. Conditional deletion of *Atp6v0a2* exons 12–14 occurs in hematopoietic stem cells in Vav1^Cre^-crossed mice, DN2 thymocytes in Lck^Cre^-crossed, and in DP thymocytes in CD4^Cre^-crossed. Cre^+^a2V^fl/fl^ mice were compared to Cre^+^ or a2V^fl/fl^ littermates. All animals were bred and housed under pathogen free conditions, and experimental protocols were performed under approval of the RFUMS IACUC. Mice used in this study were 6–10 weeks of age. Both male and female mice were used, with no differences noted between sexes.

### qPCR

Quantitative PCR of V-ATPase isoforms was performed with SYBR green (Applied Biosystems) and the following primers: *Atp6v0a1*, 5′ GGACATGATCGACTTAGAGGCCA 3′ and 5′ ACTGCTGGGTTTTTCGCAGG 3′; *Atp6v0a2*, 5′ CTCTGTGTACACCGGCCTCA 3′ and 5′ CTGCAAAGTCCTGCTGTGCC 3′; *Atp6v0a3*, 5′ GCACCAAGCAATCCACACCA 3′ and 5′ CTCAGACAGCTGGGCATGGG 3′; *Atp6v0a4*, 5′ TGCCGGGGAAACGTGTACTT 3′ and 5′ AGCACGAAACCCGTCACAGA 3′. Measurement of Notch1 target genes utilized the following primer pairs: *Dtx1*, 5′ GTGTGCCGCAAGACCAAGAA 3′ and 5′ GAGTACATGGCCACCAGGCA 3′; *Ptcra*, 5′ TAGCTCCTGGCTGCAACTGG 3′ and 5′ GCATCGAGGACCAGGCAAAC 3′; *Hes1*, 5′ TGTCAACACGACACCGGACA 3′ and 5′ TGGAATGCCGGGAGCTATCTT 3′. *Notch1* was measured with the primer pair 5′ GTGCCTGCCCTTTGAGTCTT 3′ and 5′ GCGATAGGAGCCGATCTCATTG 3′. Expression analysis was performed with GENEX (BioRad).

### Antibodies

The following antibodies were obtained from BD Biosciences or BioLegend: APC anti-CD4 (GK1.5), CD11c (N418), CD24 (M1/69), CD44 (IM7), and TCRβ (H57-597); APC-Cy7 anti-CD4 (RM4-5), CD19 (6D5), and CD25 (PC61); FITC anti-CD11b (M1-70), CD44 (IM7), CD62L (MEL-14), and TCRγδ (UC7-13D5); PE anti-CD117 (2B8), CD5 (53-7.3), CD8α (53-6.7), F4/80 (BM8), and CD25 (PC61); PE-Cy7 anti-CD3ε (145-2C11) and CD127 (SB/199); PerCP-Cy5.5 anti-CD4 (GK1.5), CD45.2 (104), and TCRβ (H57-597); Pacific Blue anti-CD4 (GK1.5), CD8α (53-6.7), NK1.1 (PK136), and Sca-1 (D7); BV421 anti-CD19 (1D3) and CD8α (53-6.7); BV605 anti-CD4 (GK1.5), CD69 (H1.2F3), Ly6G (1A8), and TCRγδ (GL3).

### Flow Cytometry and Cell Sorting

Single cell suspensions were treated with Fc block (2.4G2) for 20 min at room temperature, and then stained in FACS buffer containing the antibody cocktail for 30 min at 4°C. Annexin V staining was performed according to the manufacturer's protocol (BD Biosciences). Stained cells were washed in FACS buffer and sorted on a FACS Aria (Becton Dickson) or fixed in 4% PFA and analyzed on an LSR-II (Becton Dickson). FCS files were further analyzed via FlowJo software (Tree Star, Inc).

### Bone Marrow Chimeras

Bone marrow from Cre^+^a2V^fl/fl^ (Vav^Cre^, Lck^Cre^, or CD4^Cre^; CD45.2^+^) and C57BL/6J (CD45.1^+^) donor mice was collected and RBCs were lysed in ACK buffer. The remaining cells were washed in PBS, incubated with anti-Thy1.1 media, and separated on an AutoMACS Pro (Miltenyi Biotech) to remove residual T cells. 1 × 10^6^ of the remaining cells were injected intravenously into sub-lethally irradiated Rag1^−/−^ recipient mice at a 1:1 ratio of CD45.2:CD45.1 cells. Frequency of donor-derived hematopoietic cells including thymocytes and major sub-populations were analyzed 6 weeks post reconstitution.

### OP9 Cultures

OP9-DL4 cells were generously provided by J.C. Zuniga-Pflucker (University of Toronto, Toronto, Canada) and used as previously described for OP9-DL1 cells (18). Sorted populations of mixed DN2 and DN3 cells were seeded at 1 × 10^4^ cells/well onto sub-confluent stromal cell monolayers in 24-well plates and cultured for 8 days in the presence of 5 ng/mL Flt3L (PeproTech). Cells were passaged at 5 days in fresh media and cytokines onto new monolayers. Absolute counts were determined by size exclusion on an Accuri C6 flow cytometer (BD Biosciences).

### Western Blotting

Whole cell lysates were prepared in RIPA buffer and a protease inhibitor cocktail. Nuclear and cytoplasmic fractions were obtained by first incubating sorted cells in low salt buffer (10 mM HEPES, 10 mM KCl, 0.2 mM EDTA, 1 mM DTT), followed by centrifugation and collection of the cytoplasmic fraction supernatant. The remaining pellet was washed twice and the resuspended in a high salt nuclear extraction buffer (20 mM HEPES, 400 mM NaCl, 2 mM EDTA, 1 mM DTT). Lysates were resolved by SDS-PAGE in a 6–15% gradient gel and transferred to a nitrocellulose membrane. Membranes were blocked with 5% non-fat milk in TBST (10 mM Tris, pH 8.0, 150 mM NaCl, 0.5% Tween 20) for 60 min, then incubated with primary antibodies for 12 h at 4°C. Membrane was washed with TBST three times and incubated with HRP-conjugated secondary antibodies for 60 min at room temperature. The membrane was washed three times and developed using the ECL system. Primary antibodies used included cleaved Notch1 (D3B8, 1:1000), β-Actin (13E5, 1:2000), polyclonal SMAD2/3 (1:1000), and P-SMAD2 (E8F3R, 1:1000), all purchased from Cell Signaling Technologies, and polyclonal TBP (1:1000) from Proteintech.

### Statistical Analysis

All data are represented as mean ± SEM unless otherwise stated. Unpaired two-tailed *t*-test was used to calculate statistical significance, with the significance level set at *p* < 0.05. OP9 cocultures were analyzed by two-way ANOVA with post hoc Holm-Šídák method of multiple comparisons. Analysis was performed using SigmaPlot (Systat Software).

## Results

### Deletion of a2V Results in Peripheral Leukopenia Primarily Confined to CD4^+^ and CD8^+^ αβ T Cells

**Figure 1:**
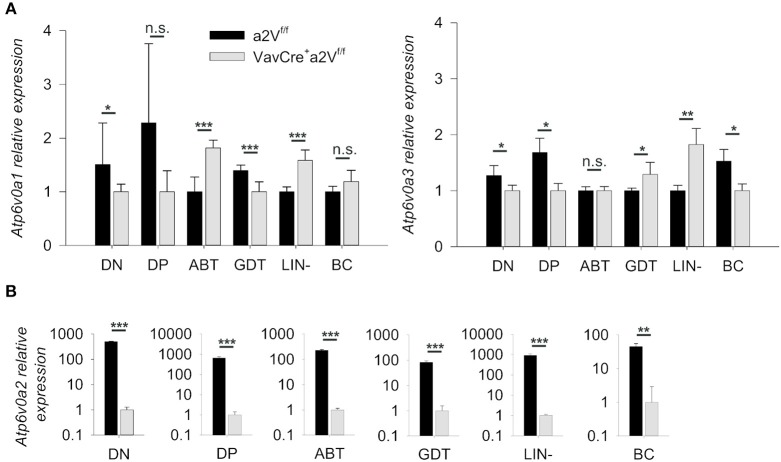
Quantification of V-ATPase isoform mRNA expression. **(A)** *Atp6v0a1* (a1V) and *Atp6v0a3* (a3V) isoform expression levels relative to GAPDH in sorted leukocyte populations from a2V^fl/fl^ and Vav^Cre^a2V^fl/fl^ thymuses (DN and DP) or spleens (ABT, GDT, Lin-, and BC). **(B)** *Atp6v0a2* (a2V) isoform expression relative to GAPDH and β-Actin in sorted populations of splenocytes and thymocytes. *Atp6v0a4* (a4V) expression was not detectable in any populations. ABT, αβ T cells (CD3^+^TCRβ^+^); BC, B cells (CD19^+^); DN, double negative thymocytes (CD3^−^CD4^−^CD8^−^); DP, double positive thymocytes (CD3^+^CD4^+^CD8^+^); GDT, γδ T cells (CD3^+^TCRγδ^+^); Lin, mixed lineage splenocytes (CD3^−^CD19^−^). Expression data was pooled from three independent experiments (total *n* = 8) and representative of 2 other independent experiments. Numeric data represents means ± SEM. n.s., no significance, ^*^p ≤ 0.05, ^**^p ≤ 0.01, ^***^p ≤ 0.001.

To conditionally delete a2V in hematopoietic cells, we crossed a2V^fl/fl^ and Vav^Cre^-recombinase expressing strains, then monitored V-ATPase *a* subunit isoform expression (*Atp6v0a1*, a1V; *Atp6v0a2*, a2V; *Atp6v0a3*, a3V; and *Atp6v0a4*, a4V) to confirm specific deletion of a2V within hematopoietic cells of Cre-expressing mice (Figures 1A,B). As expected, sorted splenocytes (ABT, GDT, Lin-, and BC) as well as sorted DN and DP populations from the thymus all displayed an ablation of a2V mRNA transcripts as compared to control mice. a1V and a3V were present in all assayed cell populations, with some small differences in expression levels in a2V-deficient cells as compared to a2V-sufficient controls. a4V expression was not detected in any assayed leukocyte cell population.

**Figure 2:**
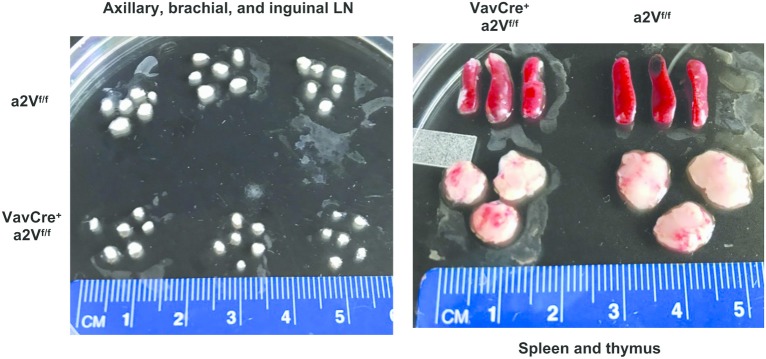
Reduced lymphoid organ size in Vav^Cre^a2V^fl/fl^ mice. Lymph nodes, spleens, and thymuses of Vav^Cre^a2V^fl/fl^ mice are grossly reduced in size. The difference is most pronounced in the T cell dominated organs of lymph nodes and thymuses (*n* = 3 each group).

**Figure 3:**
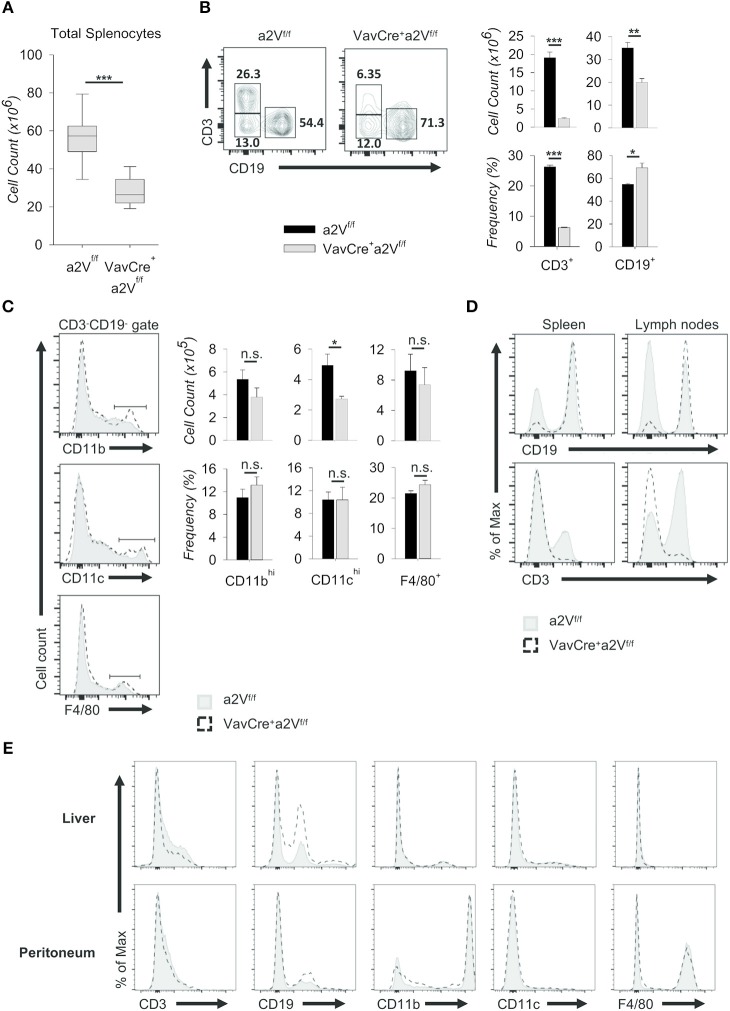
Characterization of peripheral lymphopenia in Vav^Cre^a2V^fl/fl^ mice. Cells from spleens, lymph nodes, livers, and peritoneal cavities of a2V^fl/fl^ and Vav^Cre^a2V^fl/fl^ mice were isolated, counted by Trypan blue exclusion on a hemocytometer or by size exclusion on a BD Accuri C6, and analyzed by flow cytometry **(A–E)**. **(A)** Absolute number of cells isolated from a2V^fl/fl^ and Vav^Cre^a2V^fl/fl^ spleens (*n* = 9 each group). Data are pooled from three independent experiments and representative of seven total experiments. **(B)** Representative flow plots and quantification by frequency and absolute number of T (CD3^+^) and B (CD19^+^) cells. **(C)** Representative flow plots and quantification by frequency and absolute number of major myeloid populations within the CD3^−^CD19^−^ gate. **(D)** Flow cytometric analysis of T and B cell population shifts in a2V^fl/fl^ and Vav^Cre^a2V^fl/fl^ spleens and lymph nodes. **(E)** Frequencies of major cell subsets in non-lymphoid tissues of liver and peritoneum. Numbers in flow plots represent the frequency of each gate. Numeric data represents means ± SEM. Data in histograms are normalized to the mode. Unless otherwise noted, all data are representative of at least three independent experiments with *n* ≥ 3. n.s., no significance, *p ≤ 0.05, **p ≤ 0.01, ***p ≤ 0.001.

Upon deletion of a2V we observed gross phenotypic changes consistent with a severe peripheral leukopenia, particularly in the peripheral lymph nodes and thymuses (Figure 2). While visual observation of relative spleen size was consistent between a2V-sufficient and -deficient animals (Figure 2), we still observed a significant reduction in splenic cellularity (Figure 3A). Within the spleen, the total numbers of both CD3^+^, and CD19^+^ lymphocytes were reduced with a2V deletion, but only the CD3^+^ T cell compartments were substantially reduced by frequency (Figure 3B). Additionally, though numbers of CD11b^+^, CD11c^+^, and F4/80^+^ myeloid lineages trended lower in Vav^Cre^a2V^fl/fl^ mice (Figure 3C), except in the case of CD11c^+^ cells this was not found to be statistically significant. Furthermore, these populations remained largely unchanged by our analysis of cellular frequency (Figure 3C), which included several major splenic myeloid subsets (Figures S1A,B). Therefore, a2V deletion appears to predominately affect the presence of CD3^+^ T cells in the periphery. We also hypothesized that the moderate a2V-driven decreases observed in the overall numbers of B cells and dendritic cells were directly due to the absence of T lymphocytes.

Consistent with the finding that lymph nodes from Vav^Cre^a2V^fl/fl^ mice displayed a greater reduction in size than spleens when compared to a2V^fl/fl^ control animals (Figure 2), the overall shift in CD3^+^ and CD19^+^ populations within the lymph nodes was more pronounced than in the spleen (Figure 3D). We speculated that in the lymph nodes, a reduced presence of T cells creates a void presumably more frequently occupied by B cells than by other lineages as in the spleen. Similar findings were noted in the non-lymphoid tissues of liver and peritoneum while no differences were observed in myeloid cells (Figure 3E). Together, these data suggest a global leukopenia driven primarily by cells within the CD3^+^ T cell compartment.

**Figure 4:**
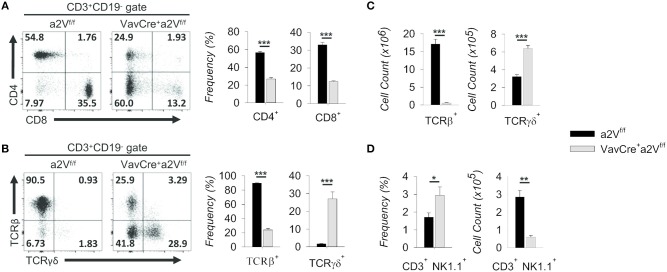
Characterization of splenic T cell compartment in Vav^Cre^a2V^fl/fl^ mice. **(A)** Flow cytometric analysis and quantification of CD3^+^ splenocytes on the basis of CD4 and CD8 surface expression. **(B)** Flow cytometry and quantification of CD3^+^ gated splenocytes on the basis of TCRβ and TCRγδ surface expression. **(C)** Absolute numbers of TCRβ and TCRγδ populations in a2V^fl/fl^ and Vav^Cre^a2V^fl/fl^ spleens. **(D)** Frequency and absolute numbers of NK1.1^+^ splenocytes within the CD3^+^CD4^−^CD8^−^ gate. Numbers in flow plots represent the frequency of each gate or quadrant. Numeric data represents means ± SEM. Unless otherwise noted, all data are representative of at least three independent experiments with *n* ≥ 3. n.s., no significance, *p ≤ 0.05, **p ≤ 0.01, ***p ≤ 0.001.

We next further examined the splenic CD3^+^ compartment and found in Vav^Cre^a2V^fl/fl^ mice an approximately 50 and 60% mean frequency reduction of CD3^+^CD4^+^ and CD3^+^CD8^+^ T cells, respectively, along with an increased frequency of CD3^+^CD4^−^CD8^−^ cells (Figure 4A). The decrease in CD4/CD8 frequency is independent of a significant change in the overall splenic CD4:CD8 ratio (1.72 in a2V^fl/fl^ controls and 2.18 in Vav^Cre^a2V^fl/fl^ mice; *p* = 0.113), suggesting a near equal reduction in these populations and normal homeostatic control of peripheral T cell populations in Vav^Cre^a2V^fl/fl^ animals. Moreover, the mean frequency of total TCRβ^+^ cells was reduced by nearly 75%, while γδ T cells increased 150% (Figure 4B). By number, these shifts corresponded to a >95% reduction in TCRβ^+^ cells, while TCRγδ^+^ cell counts nearly doubled as compared to control (Figure 4C). Although this could be due to a developmental bias toward the γδ T lineage, we speculated that because γδ T cells emerge first and with greater frequency early in life (19), their increased number in Vav^Cre^a2V^fl/fl^ mice could be the result of homeostatic proliferation within an overall lymphopenic environment, compounded by frequency enrichment. Further investigation is needed to resolve this, but from a phenotypic standpoint it is clear that γδ T cells are present in a2V-deficient mice while TCRβ^+^ T cells are nearly absent. Additionally, we found that although NK1.1^+^ cells were increased by frequency within the CD3^+^ compartment of the spleen, their total numbers were reduced by nearly 80% (Figure 4D). This is congruent with an αβ T cell deficiency, as iNKT cells arise from the thymocyte DP pool during thymic selection. Though all hematopoietic cells in Vav^Cre^a2V^fl/fl^ mice lack a2V, together these data suggest that this deficiency specifically affects the development or fitness of cells within the αβ T cell-lineage developmental pathway.

### Cell-Intrinsic Disruption of Intrathymic Development in a2V-Deficient Mice

**Figure 5:**
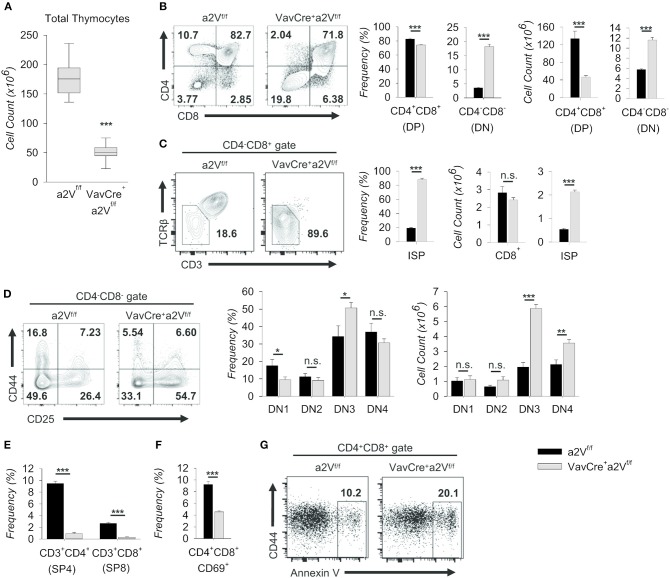
Characterization of intrathymic development in Vav^Cre^a2V^fl/fl^ mice. Thymocytes from a2V^fl/fl^ and Vav^Cre^a2V^fl/fl^ mice were isolated, counted by Trypan blue exclusion on a hemocytometer or by size exclusion on a BD Accuri C6, and analyzed by flow cytometry **(A–G)**. **(A)** Absolute number of thymocytes isolated from a2V^fl/fl^ and Vav^Cre^a2V^fl/fl^ thymuses (*n* = 9 each group). Data are pooled from three independent experiments and representative of 10 total experiments. **(B)** Flow cytometry and quantification of total thymocytes on the basis of CD4 and CD8 expression. **(C)** Flow cytometry and quantification of CD8^+^CD3^−/lo^TCRβ^−/lo^ (ISP) cells within the CD4^−^CD8^+^ gate. **(D)** Representative flow plots and quantification of DN subpopulations based on the surface expression of CD44 and CD25 (DN1, CD44^+^CD25^−^; DN2, CD44^+^CD25^+^; DN3, CD44^−^CD25^+^; DN4, CD44^−^CD25^−^). **(E)** Frequency of post-selection SP thymocytes. **(F)** Frequency of CD69^+^ DP thymocytes, representative of DP thymocytes that have begun positive selection. **(G)** Representative flow cytometric analysis of DP gated Annexin V^+^ thymocytes. Numbers in flow plots represent the frequency of each gate or quadrant. Numeric data represents means ± SEM. Data are pooled from 3 independent experiments (**B–D**, *n* = 6 each group) or are presented as a single independent experiment (*n* = 5 (Vav^Cre^a2V^fl/fl^) and *n* = 4 (a2V^fl/fl^)) and representative of at least 3 independent experiments. n.s., no significance, *p ≤ 0.05, **p ≤ 0.01, ***p ≤ 0.001.

To assess the potential consequences of a2V deletion on T cell development, we first examined thymic cell populations in experimental mice. In congruence with other lymphoid tissue, thymuses of Vav^Cre^a2V^fl/fl^ mice were also decreased in size (Figure 2) and in total cell number as compared to controls (Figure 5A). This deficiency was driven primarily by an approximately 3-fold decrease in the number of DP thymocytes (Figure 5B), although accompanied by a corresponding increase in the number and frequency of DN cells (Figure 5B). Additionally, we discovered that a majority of Vav^Cre^a2V^fl/fl^ cells in the CD4^−^CD8^+^ gate were CD3^−^ and TCRβ^−/lo^ (Figure 5C), identifying them as pre-DP immature single-positive cells (ISP) (20). This reduction in DP cells but accumulation of pre-DP DN and ISP cells within Vav^Cre^a2V^fl/fl^ mice strongly suggested a potential disruption in the DN-to-DP transition. To ascertain where this disruption might first manifest, we further examined DN subsets on the basis of CD44 and CD25 expression and found shifts in the frequencies of these subpopulations, most notably a nearly 50% increase in the frequency of Vav^Cre^a2V^fl/fl^ DN3 thymocytes (Figure 5D). Although the frequency of DN4 cells appeared slightly reduced in Vav^Cre^a2V^fl/fl^ mice, they, along with DN3 thymocytes, were significantly increased by total number (Figure 5D). This, along with the accumulation of ISP cells (Figure 5C), suggests that a2V-deficient mice have a reduced capacity to navigate developmental events surrounding the β-selection checkpoint and beyond. This phenomenon may have a metabolic basis or be related to a number of signaling pathways—including Notch1- and pre-TCR-mediated events—all of which converge at the β-selection checkpoint to drive proliferation and progression into later stages (21–23).

Similar shifts in DN and DP thymocytes are seen in mice where Notch signaling is ablated during early thymopoiesis (6, 24); however, in contrast to these studies, Vav^Cre^a2V^fl/fl^ mice were also reduced in the frequency of post-selection CD3^+^ SP4 and SP8 cells (Figure 5E). In further support of later-stage a2V influence in DP cells, the frequency of Vav^Cre^a2V^fl/fl^ CD69^+^ DP thymocytes—those which have engaged in positive selection—was also decreased (Figure 5F); and conversely, the frequency of Annexin V^+^ DP cells was roughly doubled (Figure 5G). Together, these data suggest specific disruption of αβ T cell development at multiple time points in Vav^Cre^a2V^fl/fl^ mice: a partial block during the DN to DP transition resembling an early stage Notch1 deficiency, and a near total obstruction in the primarily TCR-mediated DP-to-SP transition. The increase of Annexin V^+^ cells in the DP stage is consistent with a failure to engage in positive selection upon a2V deletion. However, the surface expression of TCRβ and various TCRα chains remained unchanged with a2V deletion (not shown), suggesting that lack of obtaining survival signals in the DP stage is not due to intrinsic differences in TCR chain expression.

**Figure 6:**
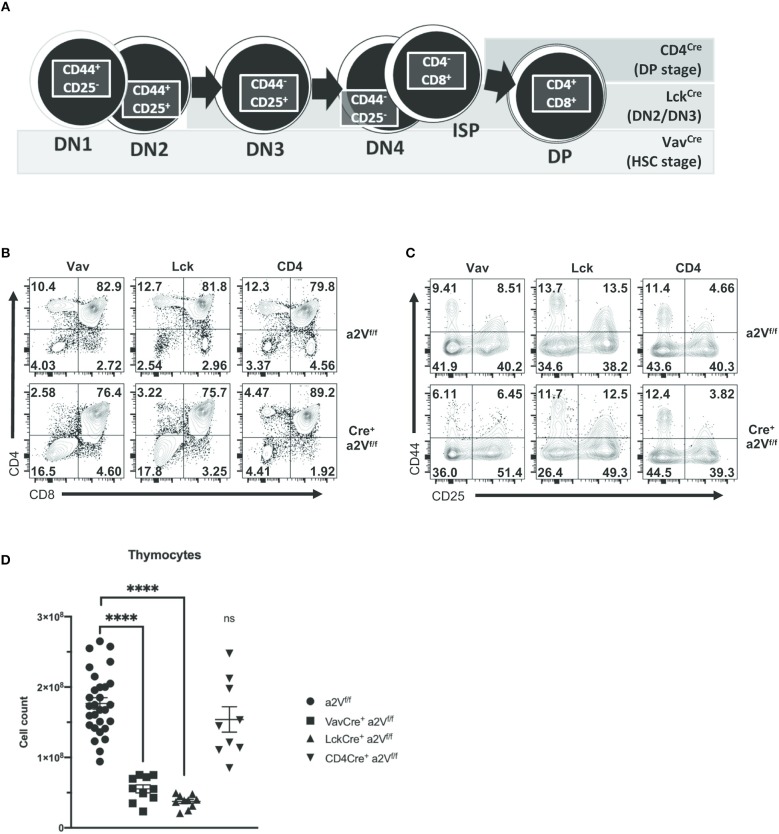
Gross characterization of thymocyte populations in a2V^fl/fl^ Cre-expressing mice. Vav^Cre^ is expressed and deletes a2V in HSC prior to T lineage commitment, while Cre-mediated deletion is restricted to developing T cells in Lck^Cre^- (deletes in DN2/DN3 stage) and CD4^Cre^- (deletes during pre-DP/DP stage) expressing strains. **(A)** Schematic showing developmental progression of DN thymocytes and its correlation with the timing of Cre expression in experimental strains. **(B)** Representative flow cytometry comparing total thymocytes in a2V KO strains and littermate controls on the basis of CD4 and CD8 surface expression. **(C)** Flow cytometric analysis of DN subpopulations in a2V^fl/fl^ and Cre^+^a2V^fl/fl^ mice on the basis of CD44 and CD25 surface expression. **(D)** Thymic cellularity of experimental strains. Numbers in flow plots represent the frequency of each quadrant. All data are representative of at least three independent experiments, except for **(D)**, which is pooled data from 3 experiments and representative of many.

Given the importance of other hematopoietic cells, such as dendritic cells, in supporting thymocyte development, we next sought to ensure the defects we observed in Vav^Cre^a2V^fl/fl^ αβ T cell development were due to dysfunctional thymocytes. To investigate whether a2V plays a cell-intrinsic role in regulating thymocyte development, we first crossed a2V^fl/fl^ mice to T cell-restricted Lck^Cre^- and CD4^Cre^-expressing strains. We observed phenotypic similarities among strains, with differences explainable through consideration of the assumed timing of Cre-recombinase expression and a2V deletion (25, 26) (Figure 6A). Phenotypically, these strains mimic the Vav^Cre^a2V^fl/fl^ phenotype in that there is a decrease in the frequency of CD3^+^ SP4 and SP8 thymocytes, and in Lck^Cre^a2V^fl/fl^ thymocytes, similar shifts in the frequencies of DN and DP populations (Figure 6B). Coinciding with our hypothesis that a2V plays a fundamental role at several points in early thymocyte development, the degree of deficiency in SP4, and SP8 populations is temporally correlated with the developmental time of a2V deletion; and furthermore, though SP4, and SP8 populations in CD4^Cre^a2V^fl/fl^ mice are still drastically reduced, the DP compartment is increased. Additionally, CD4^Cre^a2V^fl/fl^ DN subset frequencies more closely resemble a2V^fl/fl^ controls than do those in experiments performed with Vav^Cre^- or Lck^Cre^-expressing strains (Figure 6C). Given that DP cells make up the vast majority of thymocytes, it is not surprising that Lck^Cre^a2V^fl/fl^ mice, similar to Vav^Cre^a2V^fl/fl^ in their attenuated development of DP cells, are drastically reduced in total thymocyte number (Figure 6D). CD4^Cre^a2V^fl/fl^ mice, in contrast, do not differ significantly from control mice. Together, these observations lend support to a necessary and perhaps multifunctional role for a2V in coordinating both early and late intrathymic developmental events.

**Figure 7:**
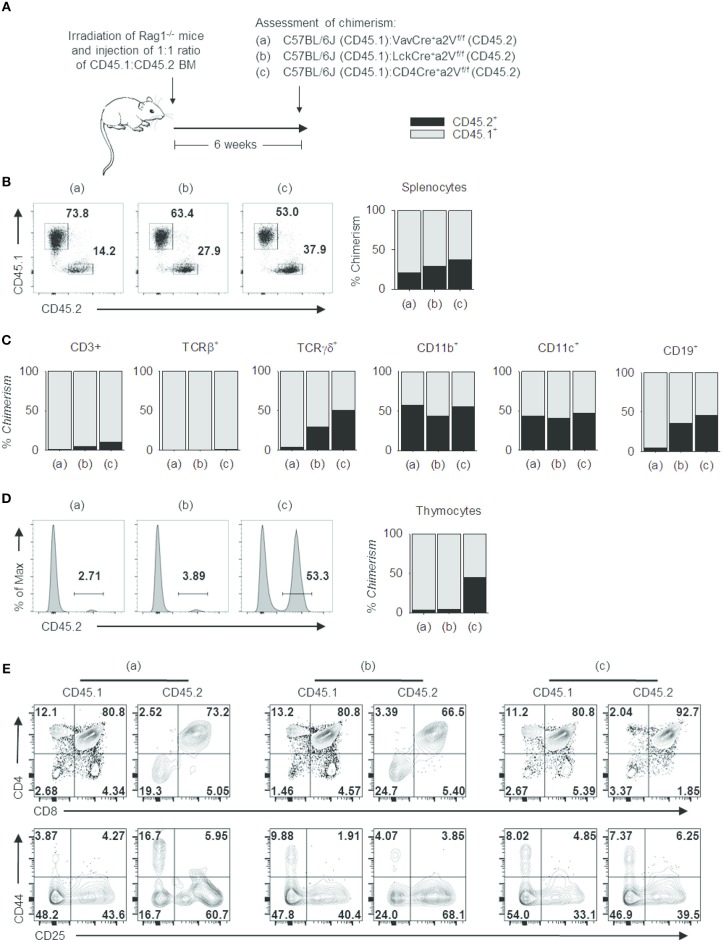
Cell intrinsic deficits in the development of a2V-deficient T cells. **(A)** Schematic representation of experimental design. **(B)** Representative flow cytometry and graphical representation of overall chimerism of splenocytes in experimental mice. **(C)** Graphical representation of % chimerism of immune cell subsets isolated from mouse spleens. **(D)** Representative flow cytometry and graphical representation of % chimerism in mouse thymuses. **(E)** Flow cytometry of thymocyte subsets in CD45.1 WT and CD45.2 a2V-KO donor cells. Data are representative of 2 independent experiments [*n* = 4, (groups a & b); *n* = 3 (group c)]. Numbers in flow plots represent frequencies within quadrants or gates.

These phenotypic similarities among Cre-expressing strains offered compelling evidence that a2V plays a cell-intrinsic role in regulating thymocyte development. We next sought to confirm this and to assess the relative developmental fitness of a2V-deficient thymocytes by preparation of mixed-bone marrow chimera mice (Figure 7A). In these chimeras, the ratio of CD45.1^+^ to CD45.2^+^ splenocytes decreased in correlation with later time points of a2V deletion (Figure 7B), driven by a CD3^+^TCRβ^+^ T cell deficiency (Figure 7C). Within the thymus, CD45.1^+^ cells are present at >95% frequency in Vav^Cre^a2V^fl/fl^ or Lck^Cre^a2V^fl/fl^ chimeras, but at equal frequency in CD4^Cre^a2V^fl/fl^ chimeras (Figure 7D). Post-selection SP4 and SP8 cells were reduced in all chimeras, but Vav^Cre^a2V^fl/fl^ and Lck^Cre^a2V^fl/fl^ donor cells were reduced in DP frequency and increased in DN frequency, a shift not observed in CD4^Cre^a2V^fl/fl^ cells (Figure 7E). Furthermore, CD4^Cre^a2V^fl/fl^ DN subpopulations closely resembled CD45.1^+^ DN cells, while Vav^Cre^a2V^fl/fl^ and Lck^Cre^a2V^fl/fl^ DN cells shifted toward increased DN3 frequency. This demonstrates that in a cell-intrinsic manner, early loss of a2V is disruptive to DN development, and that loss of a2V prior to engagement in positive selection results in a blockade of SP4 and SP8 cell development that is independent of fitness during the DN stage.

### Notch1 Signaling and Cellular Proliferation Are Reduced in a2V-Deficient Thymocytes

**Figure 8:**
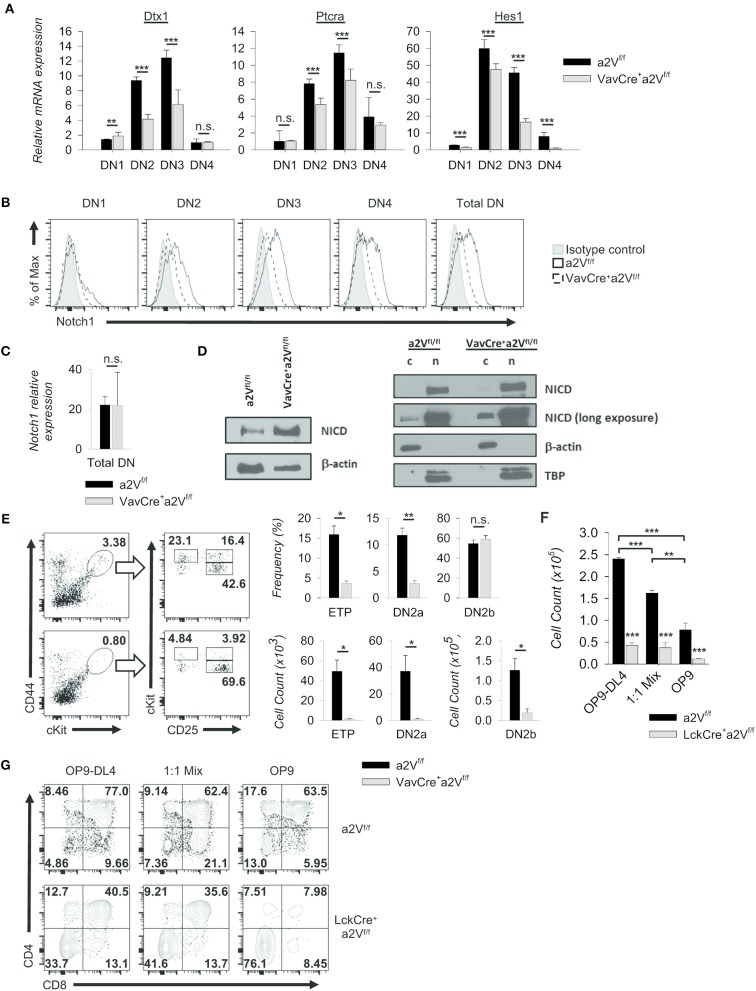
Alteration of Notch1 signaling and developmental progression. **(A)** Expression of selected Notch1 target genes relative to GAPDH in sorted DN subpopulations (CD44^+^CD25^−^ DN1; CD44^+^CD25^+^ DN2; CD44^−^CD25^+^ DN3; CD44^−^CD25^−^ DN4) (Vav^Cre^a2V^fl/fl^ and a2V^fl/fl^, *n* = 9 each group). *Dtx1* and *Ptcra* expression peak during DN3, while *Hes1* peaks during DN2. **(B)** Representative histograms of Notch1 surface staining in total DN and sorted DN subpopulations (Vav^Cre^a2V^fl/fl^ and a2V^fl/fl^; *n* = 3 each group). Data are normalized to the mode. **(C)** *Notch1* expression relative to GAPDH in total DN thymocytes. (Vav^Cre^a2V^fl/fl^ and a2V^fl/fl^; n=3 each group). **(D)** Western blot analysis of NICD in whole cell (w), cytoplasmic (c), and nuclear lysates (n). **(E)** Representative flow plots and quantification of pre- (CD25^−^CD44^hi^cKit^hi^ (ETP), CD25^+^CD44^+^cKit^hi^ (DN2a)) and post-commitment (CD25^+^CD44^+^cKit^+^ (DN2b)) thymocytes isolated from Vav^Cre^a2V^fl/fl^ (*n* = 7) or a2V^fl/fl^ (*n* = 7) mice. **(F)** Absolute counts of Lck^Cre^a2V^fl/fl^ and a2V^fl/fl^ thymocytes isolated and cultured for 8 days on stromal cell monolayers (*n* = 3 each group). **(G)** Representative flow cytometry of co-cultured DN2 and DN3 thymocyte progression toward the DP (CD4^+^CD8^+^) stage at day 5 (*n* = 3 each group). Numbers in flow plots represent the frequency of each quadrant. Numeric data represents means ± SEM. All data are representative of at least three independent experiments. n.s., no significance, *p ≤ 0.05, **p ≤ 0.01, ***p ≤ 0.001.

Notch1 signaling drives thymocyte proliferation and coordinates passage through early developmental checkpoints (27, 28). Given the similarities to a Notch1-deficient phenotype within DN cells of Vav^Cre^a2V^fl/fl^ and Lck^Cre^a2V^fl/fl^ mice, we focused on further characterization of these early events. We found that the expression of Notch1 target genes *Dtx1, Hes1*, and *Ptcra* was decreased in Vav^Cre^a2V^fl/fl^ DN thymocytes (Figure 8A), suggesting a potential reduction in developmental signals emanating from Notch1 ligation. Further, surface staining of Notch1 receptor revealed its reduced expression in all DN subsets of Vav^Cre^a2V^fl/fl^ mice, most prominently in the DN3 and DN4 populations (Figure 8B). Because expression of *Notch1* mRNA was unchanged (Figure 8C), reduced surface expression of Notch1 receptor may be due to defects in its post-translational processing or recycling at the plasma membrane. Notch signaling is highly dependent on receptor availability for ligand because it lacks an amplifying signal transduction cascade. One receptor is only capable of producing a single cleaved Notch intracellular domain (NICD), the transcriptionally active factor of the pathway. Progressive reduction in surface Notch1 and reduced target gene expression in Vav^Cre^a2V^fl/fl^ mice led us to hypothesize that generation of NICD must be reduced in a2V-deficient thymocytes. Surprisingly, western blots for cleaved Notch1 in total DN thymocytes instead revealed increased NICD; however, further examination revealed this increase was confined to the cytoplasm—the presence of NICD in nuclear lysates from Vav^Cre^a2V^fl/fl^ DN thymocytes was similar to that of a2V^fl/fl^ controls (Figure 8D). These findings are suggestive of impaired post-cleavage trafficking of NICD that may hinder translocation to the nucleus and potentiate abnormal accumulation in the cytoplasm.

In addition, we found functional evidence supporting a Notch1 defect in our examination of early thymocytes from a2V-deficient and control mice. Vav^Cre^a2V^fl/fl^ thymuses were reduced in the frequency of early thymic progenitors and pre-commitment DN2 thymocytes (Figure 8E); however, post-commitment DN2 were not significantly reduced by frequency—although absolute counts were considerably lower than control thymuses (Figure 8E). We therefore speculated that Vav^Cre^a2V^fl/fl^ thymocytes have reduced capacity to respond to the trophic effects of Notch1 signaling, but their diminished response is still sufficient to induce commitment to the T cell lineage. We next monitored responses to Notch1 signaling in post-commitment Lck^Cre^a2V^fl/fl^ or a2V^fl/fl^ thymocytes by culturing the cells on OP9-DL4 monolayers. Cellularity of all a2V^fl/fl^ wells was at least 4-fold greater than Lck^Cre^a2V^fl/fl^ wells while decreased cellularity was observed when cells were co-cultured on mixed OP9-DL4/OP9 or OP9 control monolayers (Figure 8F). Lck^Cre^a2V^fl/fl^ thymocytes appear to have a proliferative or survival disadvantage under all conditions, but in contrast to a2V^fl/fl^ cells, limiting the ratio of Notch1-expressing monolayer cells had no significant effect on total cellularity. Regardless, we speculated that Lck^Cre^a2V^fl/fl^ do not respond well to Notch1 ligation at any concentration, and therefore cannot proliferate at comparable rates to a2V^fl/fl^ cells. Because robust proliferation is required for continued differentiation into DP cells (29), we also examined the development of DP cells within co-culture wells. In all monolayer types, Lck^Cre^a2V^fl/fl^ DP and SP cells were reduced in frequency compared to controls, and furthermore this difference was exacerbated by limiting the availability of Notch ligand (Figure 8G). Together, these data corroborate *in vivo* observations and demonstrate a reduced ability of a2V-deficient thymocytes to proliferate and differentiate into DP cells, in part a result of sub-optimal Notch1 signaling.

So far, we have yet to uncover additional evidence demonstrating suboptimal surface expression of other receptors, including Notch2, Notch3, IL-7Rα, and the TCR α and β chains upon a2V deletion (Figure S2 and data not shown), suggesting that a2V does not ubiquitously influence the surface expression of every major molecule needed for thymocyte development. Though a previous report suggested a role for a2V in regulating TGFb signaling in mammary epithelial cells, we found no impact on SMAD2/3 expression or phosphorylation of SMAD2 in VavCre^+^a2V^fl/fl^ DN thymocytes (Figure S2).

## Discussion

Our report demonstrates a unique and previously undescribed role for a2V in regulating the developmental progression of thymocytes. This role appears specific to cells within the αβ T cell-lineage pathway, as we found no strong evidence of disrupted development in other lymphocytes or myeloid lineages. While we did observe a reduction in the numbers of splenic CD19^+^ and CD11c^+^ populations in Vav^Cre^a2V^fl/fl^ mice, we believe this to be a direct result of the paucity of T cells. For example, we noted that although the frequency of B cells was increased in Vav^Cre^a2V^fl/fl^ mice, these animals were unable to develop germinal centers due to a lack of follicular T helper cells (data not shown). We speculated that while B cells were generated normally, without T cell help, they are presumably impaired in their further expansion. Similarly, in the absence of interactions with T cells, DC populations are not induced to expand further. Utilization of both T cell restricted Cre strains (Figure 6) and analysis of mixed bone marrow chimeras (Figure 7) further supported a cell-intrinsic defect solely within a2V-deficient αβ T cells.

Effects on αβ T cells appear to be tied to early developmental events, as there is a reduction in cellularity of both the splenic and thymic populations. Loss of a2V manifests in part as a partial obstruction in the DN-to-DP transition, a phenotype resembling an early Notch1 deficiency in both population shifts and reduced thymic cellularity (5, 30). Previous work in Drosophila has shown that V-ATPase is required for proper activation of the Notch receptor through regulation of endosomal conditions (31, 32). Further investigation is needed to understand this relationship as it relates to mammalian T cell development, but our preliminary data demonstrating reduced Notch1 target gene expression, altered surface expression of Notch1 receptor, and abnormal processing of NICD in a2V-deficient DN thymocytes supports this notion (Figure 8). Currently, the mechanistic underpinnings of this are not yet elucidated, but we have hypothesized that loss of a2V in developing thymocytes impairs processing of the Notch1 receptor through alteration of membrane trafficking and endosomal conditions, leading to entrapment of NICD and sub-optimal signaling as well as reduced surface expression. This in turn impinges on thymocyte development during events surrounding the β-selection checkpoint, where Notch1, pre-TCR, and other signals cooperate to drive differentiation and proliferation of selected cells.

Based on our phenotypic analyses, Notch1 signaling impairment seemed to be an obvious candidate for the early arrest observed in the DN3 stage with a2V deletion. Moreover, we did not observe differences in the expression of other surface receptors, including Notch2, Notch3, IL-7Rα, and the TCR α and β chains. Regardless, it is difficult to imagine that a2V could globally influence major trafficking and internal conditions of the endosomal system yet specifically affect only Notch1. Potentially, a higher rate of Notch1 turnover in developing thymocytes could give the illusion of specificity; or more elegantly, could be the result of a2V influence on specific sorting requirements of the Notch1 receptor, imparting control over activation and release of the NICD domain (33, 34). Although V-ATPase subunits a1-a3 show nearly ubiquitous cellular expression, their specific sub-cellular localization, physiological regulation, and performance of specialized, non-overlapping functions in diverse tissues have been demonstrated (35–38). It is not a stretch to imagine that particular membrane associated dynamics of a2V are essential for the specialized sorting and fine-tuning of Notch1 signaling in a distinct tissue such as developing thymocytes, nor that this role cannot be compensated for by other *a* subunit isoforms. More general functions are performed equally well by all isoforms.

Further evidence supporting differential use and dynamic regulation of the *a* subunit isoforms—perhaps in response to loss of a2V—can be seen in our examination of isoform mRNA expression (Figure 1). Though some cells types appear to differentially utilize specific isoforms (for example, B cells in particular express much lower levels of a2V mRNA), overall, the expression of a1V and a3V mRNA varied minimally, and even where differences were noted the fold-change was quite small. However, slight but significant upregulation of a1V in peripheral αβ T cells and a3V in peripheral γδ T cells of Vav^Cre^a2V^fl/fl^ could potentially indicate a compensatory mechanism by which these cells attempt to survive the loss of a2V. Importantly, though, any survival benefit imparted by upregulation of a1V did not appear sufficient to maintain αβ T cells numbers at a level comparable to control mice. In contrast, numbers of γδ T cells in Vav^Cre^a2V^fl/fl^ were increased despite the loss of a2V. Whether this is due to the fact that a2V is unnecessary or even deleterious to the generation of γδ T cells, or that compensatory activity of upregulated a3V is beneficial, is unknown. Further investigation into the dynamics of V-ATPase isoform expression and localization during the critical periods surrounding T cell lineage choice may shed light on this question.

Progression of progenitor cells from the DN1 to later stages is dependent on a confluence of factors that sequentially restrict and redirect the divergence of other lineages during development (39, 40). γδ T cells arise during the DN2 and DN3 stages of intrathymic development, ostensibly prior to β-selection, while NKT cells emerge from the DP pool following rearrangement of TCRβ and α chains (41, 42). Because we found disruptions in a2V-deficient αβ T cell development during and through these checkpoints (Figures 5–7), including a reduction of peripheral iNKT numbers (Figure 4), yet γδ T cells were normally present in the periphery of Vav^Cre^a2V^fl/fl^ (Figure 4), we hypothesized a specific dual-stage role for a2V in regulating both the DN-to-DP transition as well as selection of DP thymocytes.

Focusing first on the early events, our data seems to point toward a specific disruption surrounding events of β-selection. While we found evidence strongly supporting a Notch1 defect in Vav^Cre^a2V^fl/fl^ (Figure 8), the convergence of multiple pathways and a dependence on robust proliferation for continued development of both pre- and post-β-selection thymocytes suggests that other factors, possibly even metabolic regulation, may also be in play (29, 43, 44). Notably, a recent report showed that mTOR activity differentially controls the balance of glycolytic and oxidative metabolism in response to changing metabolic demands during early T cell development (23). This balance directly influences ROS within developing cells and in turn influences lineage choice, with preferential use of oxidative metabolism leading to increased generation of γδ T cells. In addition, loss of mTORC1 signaling impaired proliferation, attenuating the further differentiation and development of post-β-selection thymocytes. The resulting DN-to-DP transitional phenotype in these mice is remarkably similar to the one found in Vav^Cre^a2V^fl/fl^ and Lck^Cre^a2V^fl/fl^ mice, and our mice also have a higher prevalence of γδ T cells. Considering that several reports have found a close association between V-ATPase and mTOR signaling in controlling metabolic outcomes (45, 46), it is quite possible that the phenotype in a2V-deficient mice is also associated with impaired mTOR signaling and downstream metabolic activity. Further experiments are planned to characterize the differential use of specific metabolic pathways in the presence or absence of a2V and may point toward an mTOR-related mechanism.

Compounding examination of the DN3 stage and β-selection is that there exists a differential requirement for Notch1 signaling in pre- and post-β-selection cells. Cooperation between Notch1 and pre-TCR signaling has been shown to be required for passage through β-selection (47), although the necessity of Notch1 signaling then fades as cells further mature. Because a2V-deficient thymocytes are also largely unable to navigate the TCR-mediated selection checkpoint and progress to the SP stage (Figures 5–7), we have hypothesized that a2V is also important in TCR-mediated events, independent of its effects during the DN stages. However, because of the cooperation between Notch1 and pre-TCR at β-selection, we cannot completely rule out TCR-mediated effects during this period.

Utilization of OP9-DL4 co-cultures will be beneficial in exploring this problem. Though our limiting-Notch1-ligation co-culture experiments with Lck^Cre^a2V^fl/fl^ cells showed a reduction in cellularity as compared to control cells (Figure 8F), this alone does not point to a definitive Notch1 mechanism. Reduced development of DP cells in response to limited Notch1 ligation offers some further support for a mechanism of reduced proliferation driving the defect, but again the influence of pre-TCR and metabolism associated mechanisms cannot be ruled out. It should also be noted here that Lck driven Cre expression has been shown to affect thymocyte survival in a copy number dependent manner (26). While we have not observed significant differences in cellularity or frequencies of major thymocyte subpopulations when comparing the Lck^Cre^a2V^+/+^ and a2V^fl/fl^ strains, we have not analyzed this in the OP9 coculture system. Thus, because we used a Cre^−^ control, it is possible that some of the reduction in cellularity observed in Lck^Cre^a2V^fl/fl^ wells is due to Cre toxicity. Further experiments utilizing a Cre^+^ control and seeding pre- and post-β-selection cells independently will provide a more definitive answer to all these questions, and further utilization of the OP9 *in vitro* system will give us the ability to perform overexpression and knock-down experiments that more tightly control the multiple variables in question.

Mechanistically, there is still much to uncover regarding the role of a2V in coordinating T cell development, largely due to the complex regulatory network involved in orchestration of such a critical process. However, the phenotype presented herein offers tantalizing clues to discovering the nature of this defect. Both Notch1 signaling as well as proliferative metabolic pathways have emerged as potential avenues of investigation, as have TCR-mediated pathways. The transcriptional regulation of T cell development has largely taken center stage in the field, but it remains imperative to understand how the intrinsic cell biology of developing cells works to collect, coordinate, and disseminate both environmental and nuclear signals to ultimately shape their developmental outcome. Our phenotype demonstrates that a2V plays a critical role in this regard, potentially affecting multiple pathways as a central regulator of T cell development.

## Ethics Statement

This study was carried out in accordance with the recommendations of the Institutional Animal Care and Use Committee (IACUC) at Rosalind Franklin University of Medicine and Science. The protocol was approved by the IACUC.

## Author Contributions

TP designed and performed the experiments, analyzed data, and wrote the manuscript. MJ performed foundational experiments. KB provided intellectual and material support and edited the manuscript. JR conceived of the study, performed experiments, analyzed data, and edited the manuscript.

### Conflict of Interest Statement

The authors declare that the research was conducted in the absence of any commercial or financial relationships that could be construed as a potential conflict of interest.

## Supplementary Material

Figure S1 Flow cytometry analysis of selected myeloid subsets. **(A)** Representative flow and **(B)** quantification of major functional myeloid subsets. RPMac, red-pulp macrophages (CD11b^lo^CD11c^lo^F4/80^+^); Mac, conventional macrophage (CD11b^hi^F4/80^+^); InfMo, inflammatory monocyte (CD11b^hi^Gr-1^+^); MoDC, monocyte derived dendritic cell (CD11c^hi^CD11b^+^F4/80^+^). n.s. = no significance. ^*^*p* < 0.05.

Figure S2 Analysis of TGFβ signaling components. Western blots of SMAD2/3 and P-SMAD2 in cytoplasmic and nuclear lysates from sorted DN thymocytes.
