# Supplementary figures and images for: Conditional Deletion of the V-ATPase a2-Subunit Disrupts Intrathymic T Cell Development
Source: Front Immunol. 2019 Aug 13;10:1911. doi: 10.3389/fimmu.2019.01911 (PMC6700305; doi:10.3389/fimmu.2019.01911)

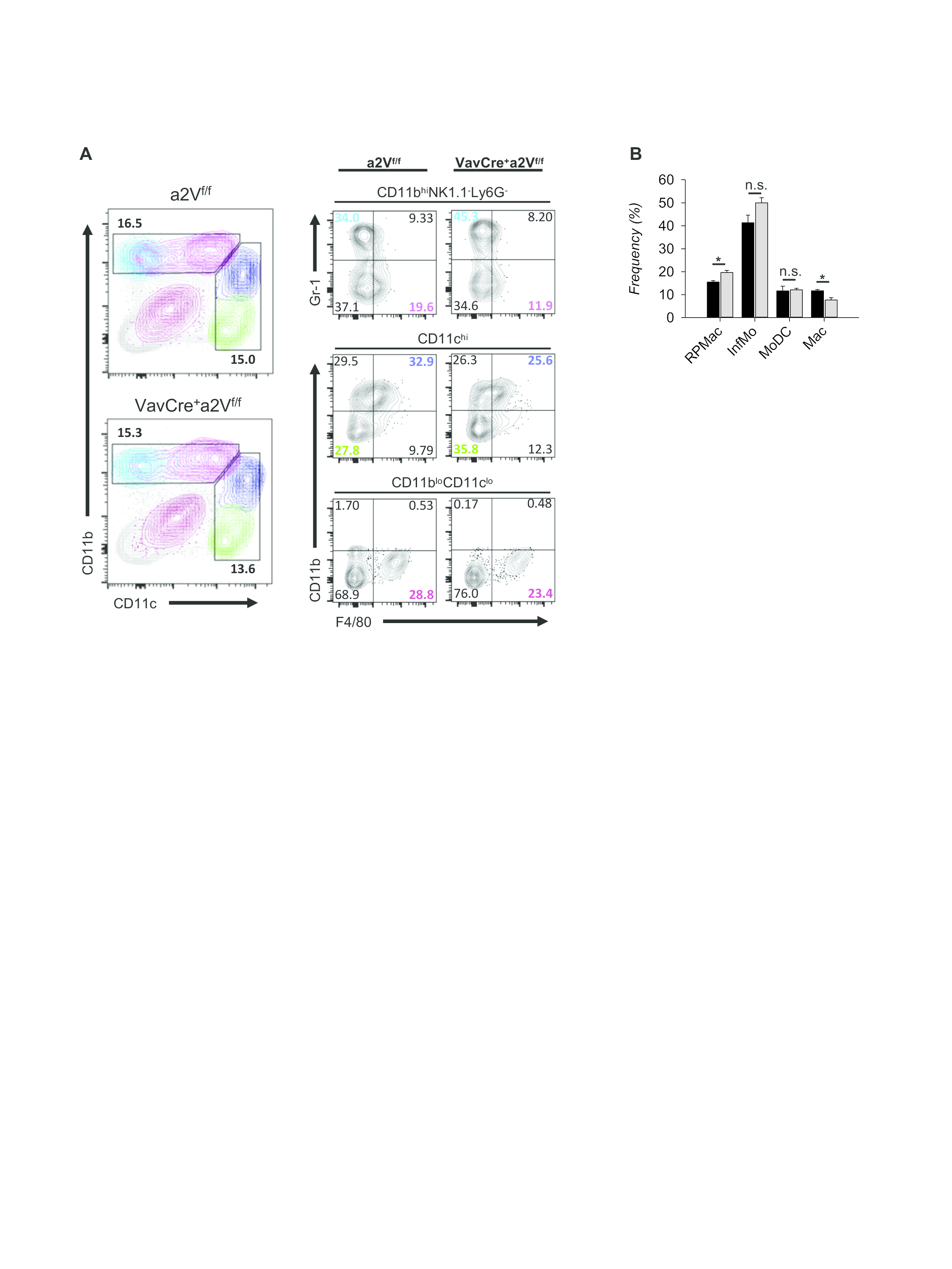

Supplement: Figure S1 — Flow cytometry analysis of selected myeloid subsets. (A) Representative flow and (B) quantification of major functional myeloid subsets. RPMac, red-pulp macrophages (CD11bloCD11cloF4/80+); Mac, conventional macrophage (CD11bhiF4/80+); InfMo, inflammatory monocyte (CD11bhiGr-1+); MoDC, monocyte derived dendritic cell (CD11chiCD11b+F4/80+). n.s. = no significance. *p < 0.05. [file Image_1.tiff]

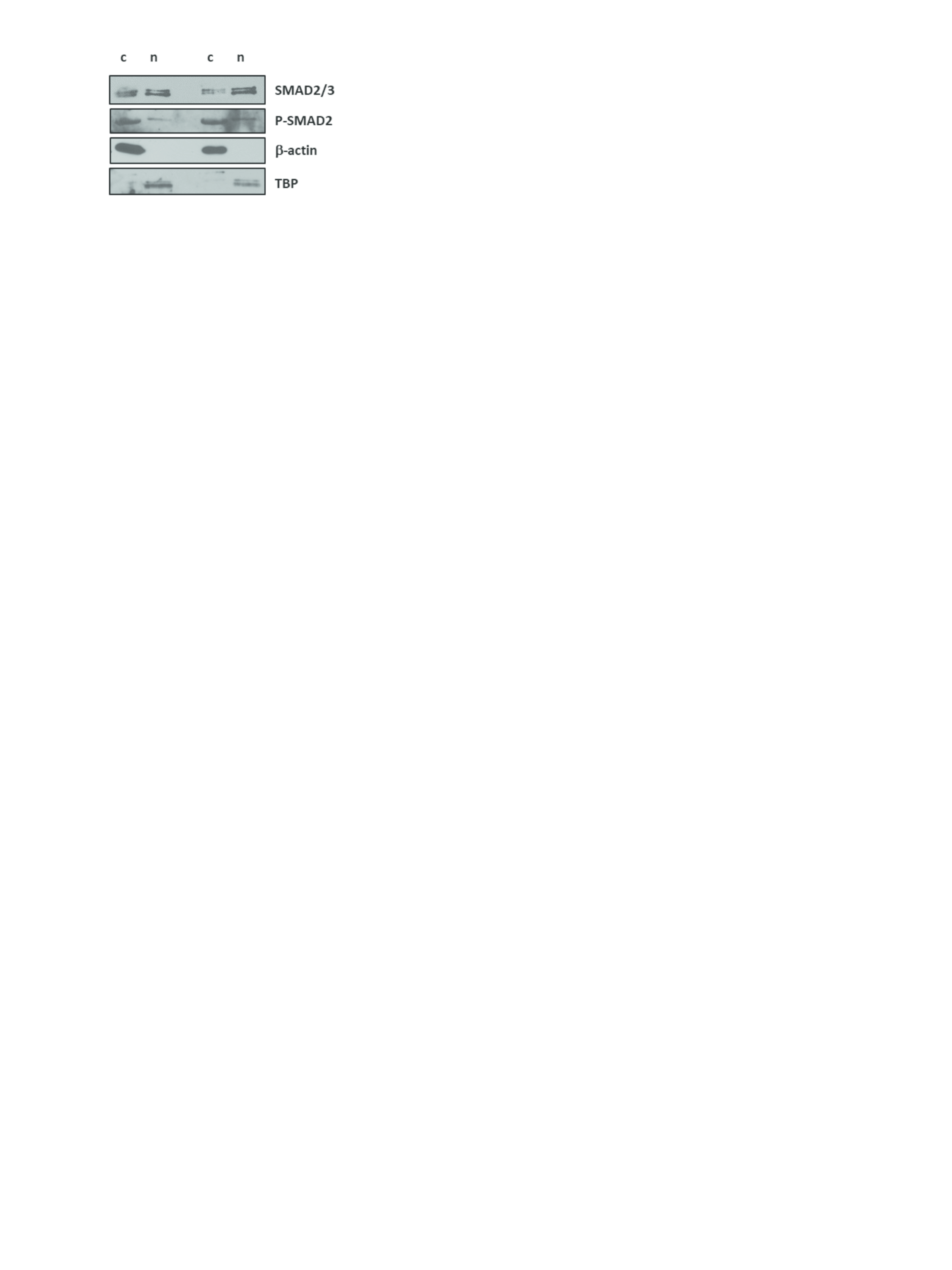

Supplement: Figure S2 — Analysis of TGFβ signaling components. Western blots of SMAD2/3 and P-SMAD2 in cytoplasmic and nuclear lysates from sorted DN thymocytes. [file Image_2.tiff]
